# Supplementary material for: (7E)-7,8-Dehydroheliobuphthalmin from Platycladus orientalis L.: Isolation, Characterization, and Hair Growth Promotion
Source: Int J Mol Sci. 2025 May 28;26(11):5189. doi: 10.3390/ijms26115189 (PMC12154219; doi:10.3390/ijms26115189)

Supplementary File

1. (7E)-7,8-Dehydroheliobuphthalmin Chemical Reference Standards and ESI+ Molecular Ion Peak Mass Spectrometry

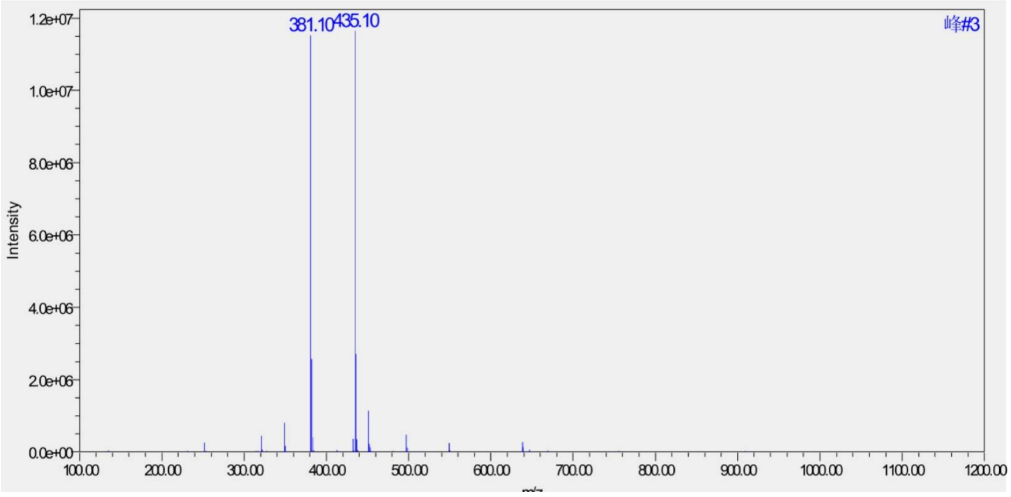

2. ^{1}H NMR Spectroscopy of (7E)-7,8-Dehydroheliobuphthalmin

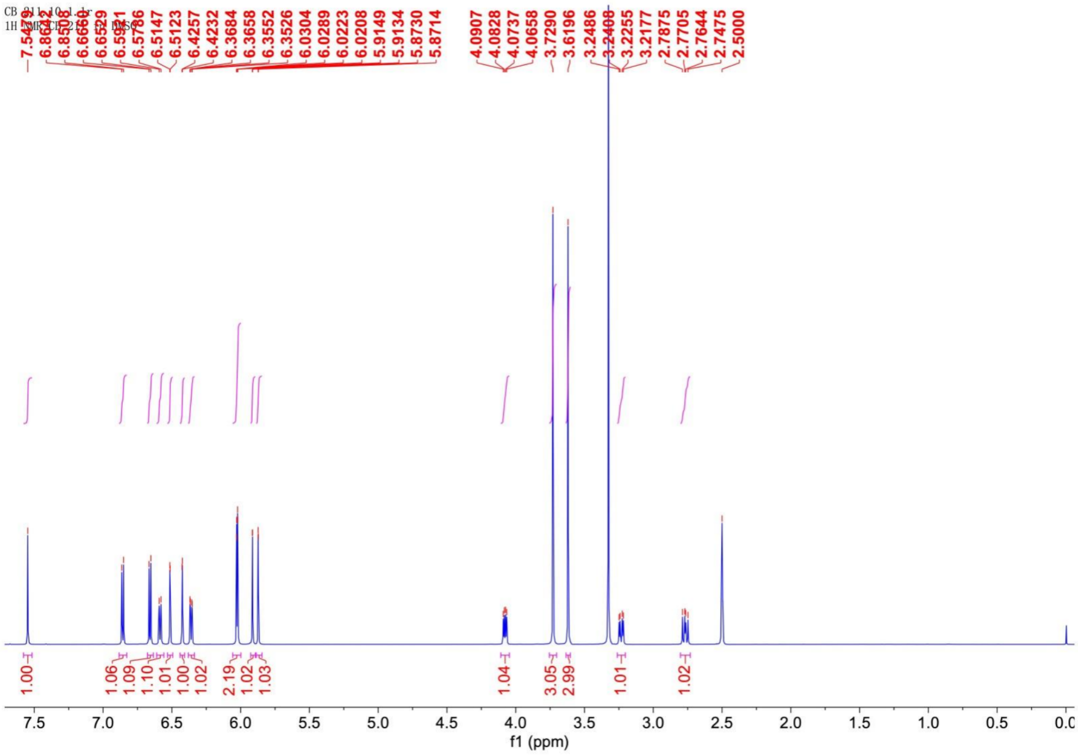

3.^{13}C NMR Spectroscopy of (7E)-7,8-Dehydroheliobuphthalmin

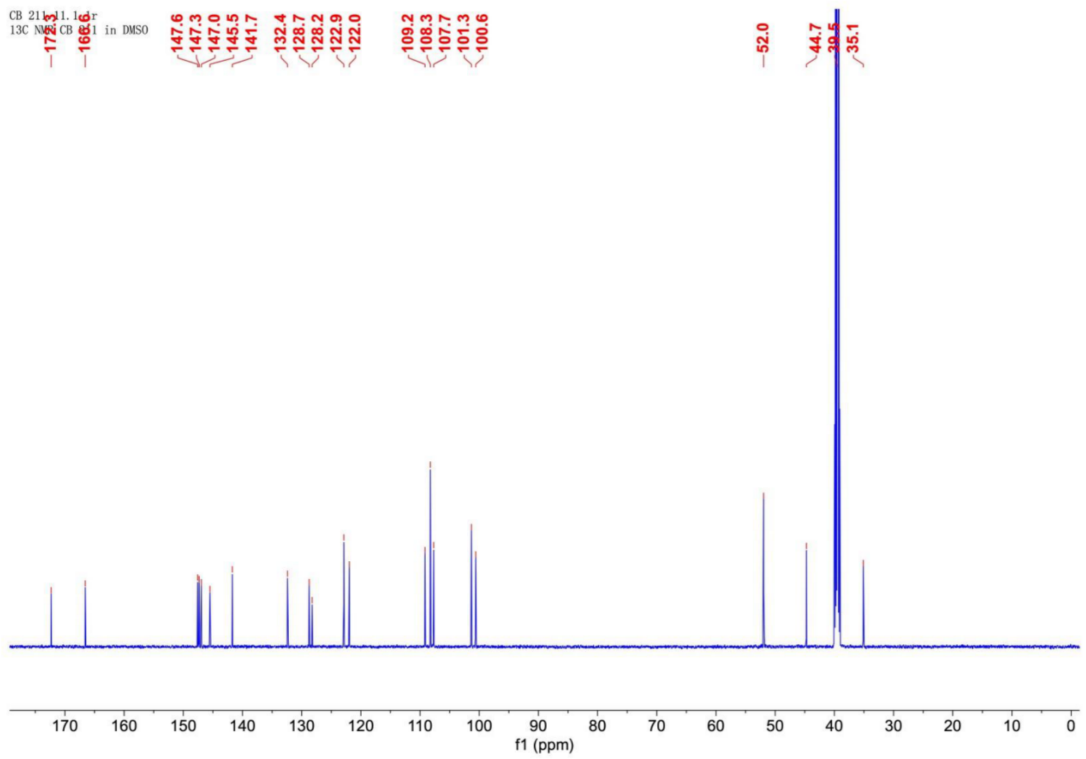

4. Infrared Spectroscopy of (7E)-7,8-Dehydroheliobuphthalmin

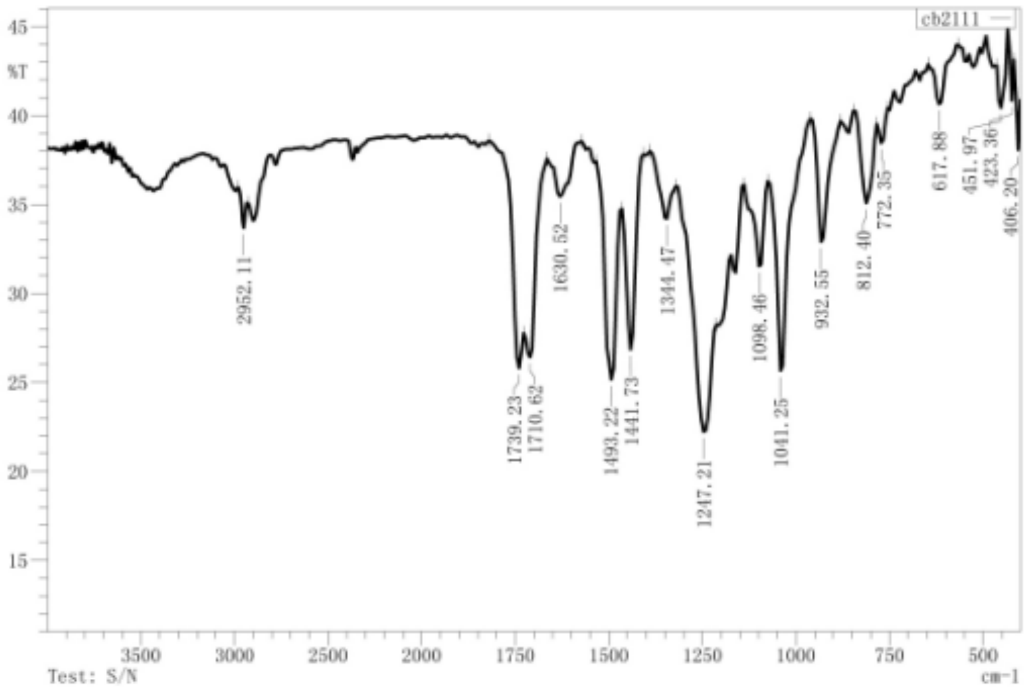

5.UV Spectroscopy of (7E)-7,8-Dehydroheliobuphthalmin

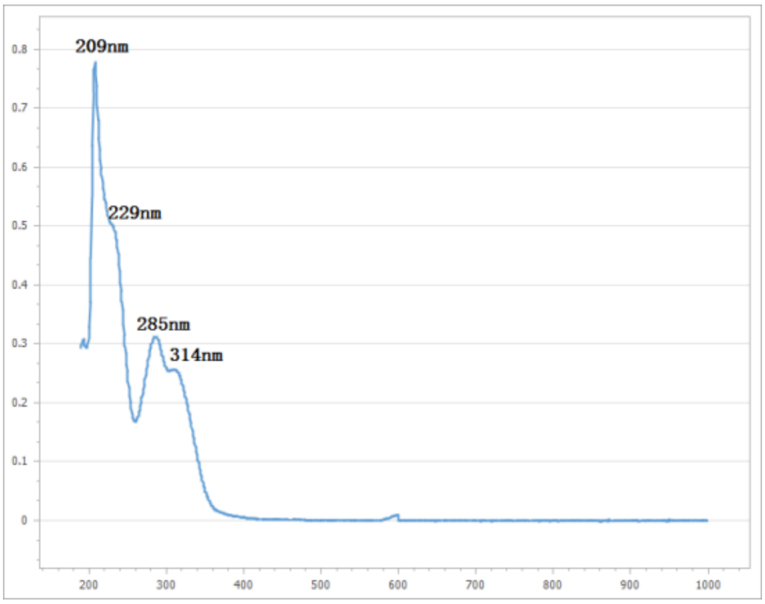

6.Optical Rotation of (7E)-7,8-Dehydroheliobuphthalmin

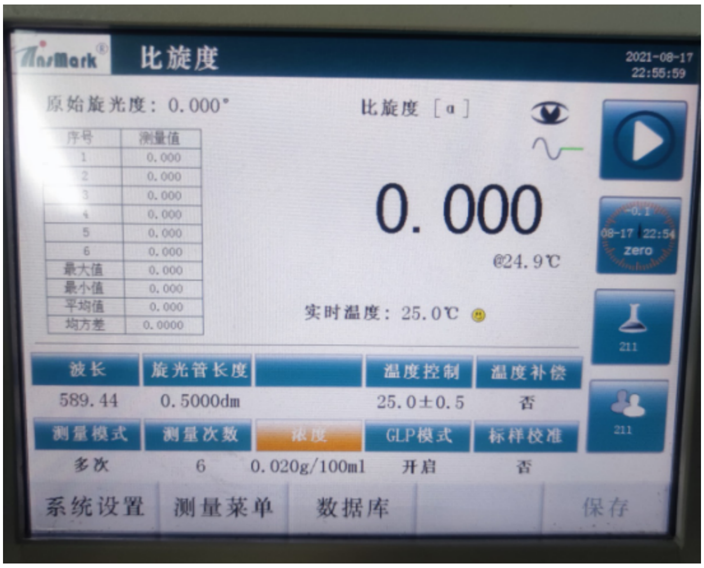

Supplement: Supplementary file 1 [file ijms-26-05189-s001.zip › ijms-3625663-supplementary.pdf]
